# Structured Habitat Attribute Extraction from Camera-Trap Images Using a Vision–Language Model: A Proof-of-Concept Study in a Karst Forest

**DOI:** 10.3390/s26196240

**Published:** 2026-10-01

**Authors:** Xiaoming Mo, Yanyan Zhao, Jifeng Long, Hui Gao, Yongjie Huang, Wenhua Li, Yang Luo, Shengyuan Liu, Yubiao Lyu, Shuangling Wang

**Affiliations:** 1 GXAS Laboratory of Forest Protection, Restoration and Ecological Utilization, Institute of Eco-Environmental Research, Guangxi Academy of Sciences, Nanning 530007, China; 2 Management Center of Guangxi Nonggang National Nature Reserve, Longzhou 532400, China; 3 Management Center of Guangxi Encheng National Nature Reserve, Daxin 532300, China; 4 Management Center of Guangxi Xidamingshan Provincial Nature Reserve, Chongzuo 532200, China

**Keywords:** camera-trap imagery, wildlife monitoring, vision–language model, habitat attribute extraction, microhabitat characterization, structured ecological data, habitat covariates, karst forest

## Abstract

Camera-trap images used in wildlife monitoring contain environmental information that is often underused. This study developed and evaluated a vision–language-model-based workflow for extracting structured visible habitat attributes from camera-trap photographs collected in a karst forest ecosystem in southern China. The workflow combined image preprocessing, schema-constrained prompting, structured JSON generation, programmatic parsing, logical-consistency validation, and automatic re-inference of invalid outputs. Seven attributes were evaluated: terrain, bare-rock visibility, vegetation layers, vegetation density, water visibility, water type, and visible human disturbance. Qwen3-VL-30B-A3B-Instruct was deployed locally, and its outputs were compared with expert-reference annotations for 102 camera-trap images. The evaluation used one model and one prompt/schema configuration and was designed to assess feasibility rather than cross-model or cross-ecosystem generalizability. All images ultimately produced valid structured records after the validation and retry procedure. Field-level exact agreement ranged from 90.20% to 99.02%. For water visibility, exact agreement was 96.08%, balanced accuracy was 97.56%, and macro-F1 was 94.20%; all 20 water-positive images were correctly identified. Water type achieved 95.10% exact agreement, with 19 of the 20 water-positive images matching the expert reference. Five experts separately verified all images using workbooks pre-populated with the reference labels while the model outputs were visible. In this non-blinded verification, Fleiss’ kappa ranged from 0.885 to 1.000 across the six estimable fields (0.974 for vegetation-layer sets treated as nominal categories); visible human disturbance had 100% observed agreement but no category variation, so kappa was not estimable. However, several reference distributions were strongly imbalanced: 96 of 102 images contained visible bare rock, 99 were assigned dense vegetation, and the validation subset contained no disturbance-positive images. The results therefore demonstrate preliminary feasibility under the evaluated conditions rather than broad generalizability. The proposed workflow provides a structured post-acquisition approach for deriving time-aligned visible habitat information from existing camera-trap imagery.

## 1. Introduction

Camera traps are widely used for non-invasive wildlife monitoring and generate large image archives containing information on species, activity, behavior, and populations [1,2,3,4,5,6]. Automated methods can now detect animals, identify species, and support large-scale image review [7,8,9,10,11,12,13]. However, most workflows remain animal-centered and make little use of environmental information recorded simultaneously in the image background, such as terrain, vegetation structure, exposed rock, surface water, and visible human disturbance.

These image-level environmental cues may complement field surveys and remote sensing. Field measurements are detailed but labor-intensive and intermittent [14], whereas remote-sensing products provide broad coverage but may not resolve understory vegetation, microtopography, or ground conditions beneath forest canopies [15,16]. Camera-trap images provide a ground-level view that is temporally synchronized with the animal record [17,18]. This is relevant in southern Chinese karst forests, where exposed bedrock, complex terrain, layered vegetation, and fragmented surface water vary over short distances [19,20,21,22]. The images do not replace environmental measurements, but they may provide a useful additional source of visible microhabitat information.

Converting this information into analyzable data is challenging because scene content is unstructured and affected by camera angle, occlusion, illumination, infrared imaging, and image quality. Habitat attributes also combine binary, categorical, and multi-label fields. A practical workflow must therefore generate records with predefined field names, allowed values, data types, and logical relationships, while retaining an uncertainty option when visual evidence is insufficient.

Vision–language models can map complex image content to semantic or structured outputs through a shared inference interface [23,24,25]. Previous camera-trap studies have used multimodal models for contextual biodiversity reporting, species recognition, and animal or scene attribute retrieval [26,27,28]. Less attention has been given to the systematic conversion of environmental backgrounds into a predefined habitat schema with programmatic validation and field-level comparison against ecological expert annotations. Such validation is necessary because structural and logical validity does not guarantee that the assigned ecological category is semantically correct.

We therefore developed a post-acquisition workflow that combines whole-image input, schema-constrained prompting, structured JSON output, programmatic parsing, logical-consistency checks, automatic re-inference of invalid outputs, and traceable storage. The schema contains seven visible habitat attributes: terrain, bare-rock visibility, vegetation layers, vegetation density, water visibility, water type, and visible human disturbance. We evaluated the workflow against expert-reference annotations for 102 camera-trap images and report field-level agreement, class-aware water metrics, imaging-mode stratification, species-associated descriptive subsets, between-reviewer agreement in expert verification, and focused review of the observed terrain and bare-rock disagreements.

This proof-of-concept study tests whether one specified workflow can convert visible scene information into standardized image-level records under the evaluated conditions. It is not a benchmark of competing vision–language models and does not estimate the separate contribution of the model, prompt, schema, or validation rules. The outputs are model-inferred visible scene characteristics, not direct measurements of physical environmental variables, and are not used here to infer animal habitat selection. Broader datasets, fully blinded de novo expert annotation, and comparative or ablation experiments remain necessary before claims of generalization or model superiority can be made.

## 2. Materials and Methods

### 2.1. Study Area and Camera Trap Photograph Data

The camera trap photographs used in this study were collected from Guangxi Nonggang National Nature Reserve, hereafter referred to as the reserve. The reserve is located in a typical karst landform region of southern China. Its vegetation is dominated by forest habitats, and the ground environment exhibits strong spatial heterogeneity [29,30]. Photographs recorded at different camera sites vary in terrain, vegetation structure, bare rock exposure, and water visibility, thus providing relatively rich natural scene samples for image-level habitat information extraction.

The reserve management center has continuously deployed infrared-triggered cameras for long-term monitoring of wildlife activity. Cameras are usually installed along animal trails, near water sources, on ridge corridors, in forest understory openings, or at other locations with relatively frequent animal activity in order to increase the probability of animal detection. These cameras can operate continuously during both day and night, recording color images during the day and infrared images at night. As a result, the dataset covers different lighting conditions and scene states.

After data collection, all camera trap photographs were manually reviewed to determine whether animals were present in the images, and identifiable animal records were organized and annotated. In this study, camera trap photographs manually confirmed to contain animals were used as the basic dataset for subsequent image-level habitat attribute extraction. The focus of this paper is the scene information presented in the entire photograph rather than individual animal targets within the image. Therefore, all subsequent analyses treated the raw camera trap photograph as the basic processing unit. To preserve complete scene information as much as possible, animal target regions were not cropped, and background regions were not manually masked at the model input stage.

To conduct manual consistency validation, this study further selected photographs of silver pheasant (*Lophura nycthemera*) and rhesus macaque (*Macaca mulatta*) from the reviewed and species-annotated dataset as sample sources. These two species had relatively good image availability in the reserve’s camera trap dataset, and their photographs covered a range of forest backgrounds and visible environmental conditions. This helped test the applicability of the proposed method across different scenes. Photographs of the two species were randomly sampled to construct a manually annotated validation subset for the subsequent assessment of field-level exact agreement between the vision–language-model outputs and expert-reference annotations. The validation subset contained a total of 102 images. The audited image-to-species mapping contained 50 images of silver pheasant and 52 images of rhesus macaque.

### 2.2. Design of the Visible Habitat Attribute Schema

To enable the environmental information contained in camera trap photographs to be transformed into structured data directly usable for subsequent analyses, we designed a standardized schema for image-level visible habitat attribute extraction, hereafter referred to as the habitat schema. The schema aimed to cover, as far as possible, visible environmental information that is both common and ecologically meaningful in camera trap photographs of karst forests, while controlling the number of fields and the complexity of interpretation in order to improve output standardization and interpretability.

Based on the scene characteristics of the study area and the interpretability of camera trap photographs, the habitat attribute fields defined in this study included terrain (terrain), bare rock visibility (bare_rock_visible), vegetation layers (vegetation_layers), vegetation density (vegetation_density), water visibility (water_visible), water type (water_type), and visible human disturbance (human_disturbance_visible). The terrain field was used to describe the visible topographic form in the image, with allowed values of flat, slope, steep, and uncertain. The bare_rock_visible field indicated whether exposed rock could be observed in the image. The vegetation_layers field recorded the visible vertical structure of vegetation in the image and could include one or more of the following elements: tree, shrub, herb, and vine. The vegetation_density field described the overall degree of vegetation cover and was categorized as dense, medium, sparse, or uncertain. The water_visible field recorded whether any visible water body was present in the image. When water was visible, the water_type field further distinguished the type of water as stream, pond, wetland, or uncertain; when water_visible was false, water_type was set to none. The human_disturbance_visible field indicated whether obvious traces of human disturbance were present. The definitions, data types, allowed values, and validation constraints for each field are shown in Table 1.

The use of a structured attribute schema rather than free-text description was mainly motivated by two considerations. First, fixed fields and constrained values help transform complex visual scenes into standardized ecological variables, facilitating subsequent statistical summarization, manual review, and cross-sample comparison [31]. Second, compared with open-ended natural language descriptions, structured outputs can substantially reduce variation in model-generated expressions and improve the parseability and reproducibility of results. At the same time, for fields with predefined uncertainty categories, the model was allowed to return conservative labels when visual evidence was insufficient, thereby reducing overinterpretation under ambiguous image conditions. To support programmatic parsing and automatic validation, field names, allowed values, and logical constraints were fixed before model inference and were used as the basis for subsequent validity checks.

### 2.3. Workflow for Habitat Attribute Extraction Based on a Vision–Language Model

In this study, Qwen3-VL-30B-A3B-Instruct [32] was used as the vision–language model for extracting visible habitat attributes from camera trap photographs. The model was called and managed through the Ollama framework and deployed in a local computing environment equipped with dual NVIDIA 4090D GPUs, enabling batch image inference.

Before model inference, each input image was inspected and proportionally resized when necessary. The resizing procedure ensured that the final image width did not exceed 1920 pixels and the final image height did not exceed 1080 pixels while preserving the original aspect ratio. Images already within both limits were retained at their original resolution. No center cropping, animal-region cropping, or manual background masking was performed because the analysis focused on environmental information across the entire scene.

**Figure 1:**
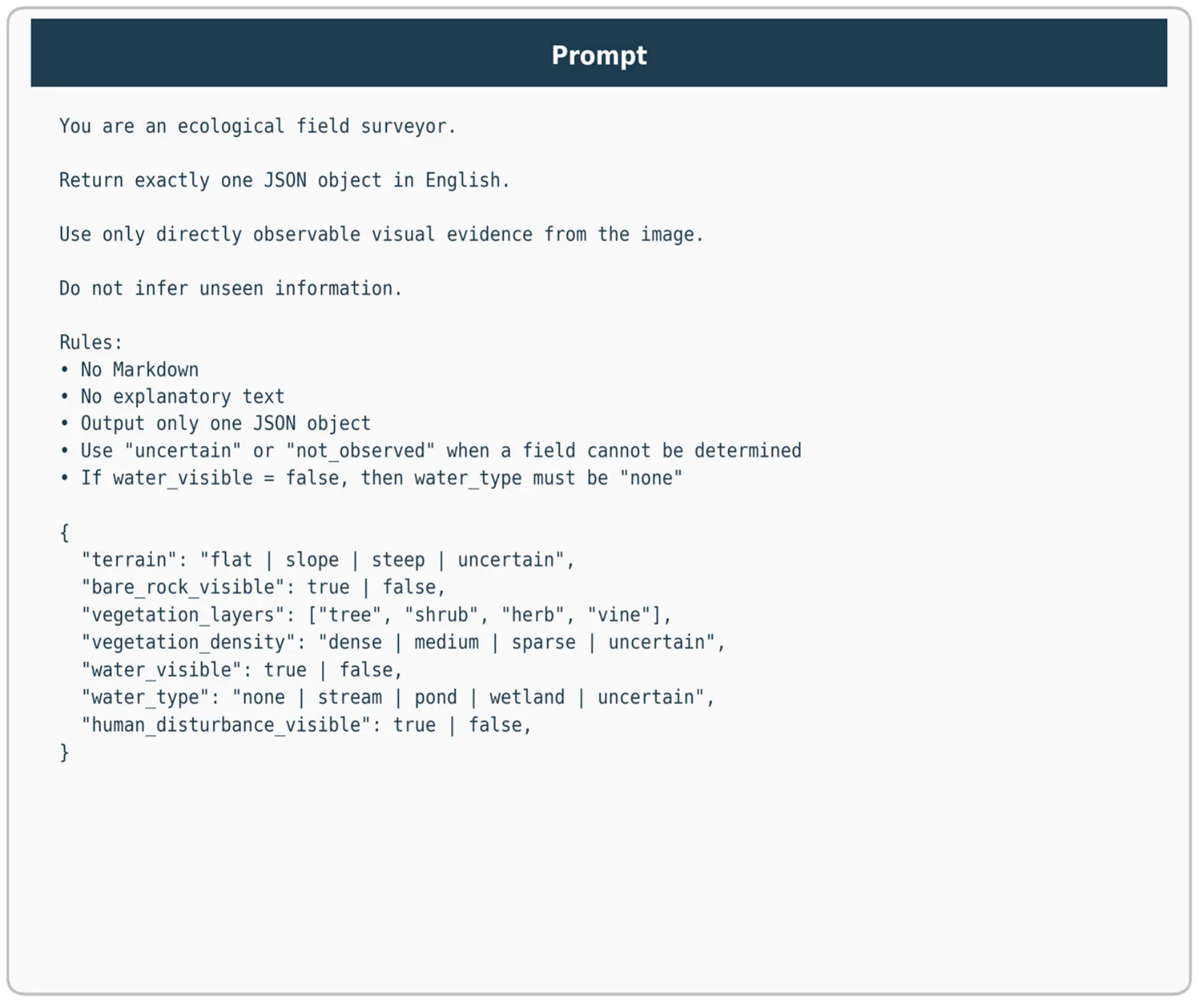
Structured prompt template and JSON output schema for visible habitat attribute extraction from camera trap photographs.

The prompt explicitly required the model to determine the visible habitat attributes in the entire camera trap photograph according to the given fields and allowed values and to output the result in standardized JSON format. The prompt specified that the model should judge terrain, bare rock visibility, vegetation structure and density, water visibility and type, and visible human disturbance based on environmental cues in the entire image. For fields with predefined uncertainty categories, the model was instructed to return a conservative uncertainty label rather than make a speculative assignment when the available visual evidence was insufficient. The output was required to strictly follow the predefined field names and allowed-value constraints and to contain only a JSON object without explanatory text. The structured prompt used for image inference and its corresponding JSON output schema are shown in Figure 1.

For inference parameter settings, generation was configured with max_new_tokens = 512, temperature = 0.1, top_p = 0.8, presence_penalty = 0.0, and streaming disabled. These settings were selected to support structured output generation while retaining sufficient capacity for scene interpretation. Each image underwent one initial inference attempt. If the request failed because of a service-call error, or if the returned output did not satisfy the JSON-format, schema, or logical-consistency requirements, the complete inference procedure was repeated up to three additional times. Thus, each image could undergo a maximum of four inference attempts. Re-inference was performed for the complete record; individual field values were not manually corrected, replaced, or imputed.

Using this workflow, model-generated content was converted into standardized structured records suitable for subsequent parsing, validation, storage, and comparison.

### 2.4. Output Parsing and Rule-Based Validation

Before entering the analytical workflow, the text results generated by the vision–language model needed to be further converted into structured records that could be computed, stored, and validated. To this end, this study programmatically parsed the model-generated results into JSON objects and performed rule-based validation according to the predefined field structure and value ranges. The purpose of this step was to improve result consistency and reduce data noise introduced by irregular output formats or drift in field meaning.

At the format parsing stage, the json.loads() function in Python 3.11.14 was first used to parse the model output. If the returned text contained Markdown code block tags, these tags were automatically removed before JSON parsing. The parsed output was then validated against the predefined schema. Validation included checking whether all required fields were present, whether the data type of each field was correct, and whether enumerated values belonged to the predefined allowed-value sets. Outputs containing missing fields, invalid data types, or values outside the allowed sets were considered invalid in their entirety and triggered a complete re-inference of the corresponding image rather than field-level correction, deletion, or imputation. For logically related fields, additional consistency constraints were applied. For example, when water_visible was false, water_type was required to be none. Where an uncertainty category had been predefined for a field, the corresponding conservative label could be used when the available visual evidence was insufficient to support a definite judgment. The validation procedure was used only to determine whether a generated record was acceptable and did not alter any semantic field value produced by the model.

If an output lacked any required field, contained an invalid enumerated value, or violated a predefined logical-consistency constraint, the complete record was marked as invalid and entered the automatic retry procedure described in Section 2.3. Up to three additional inference attempts were permitted after the initial attempt. If a record had remained invalid after all permitted attempts, it would have been retained as an abnormal record for subsequent manual review and reported as an end-to-end processing failure. In the present validation subset, all 102 images ultimately produced valid structured JSON records, and no validation image remained invalid.

After parsing and validation, the habitat attribute result corresponding to each photograph was saved as a structured record. The saved information included the raw model-generated text and the corresponding parsed and validated JSON record. Invalid outputs were regenerated rather than corrected, and the field values used in subsequent analyses were taken directly from the final model output that passed format, schema, and logical-consistency validation. This data organization preserved the model’s original judgments while facilitating subsequent manual review, error analysis, and result tracing.

The validation procedure assessed only whether a generated record satisfied the predefined format, field, data-type, allowed-value, and logical-consistency requirements. Passing these checks did not indicate that the semantic category assigned to each field was correct. Semantic agreement was evaluated separately through comparison with the expert-reference annotations.

### 2.5. Manual Annotation Validation and Performance Assessment

To evaluate the agreement between the vision–language-model outputs and expert interpretation, a manually annotated validation subset was constructed from camera-trap photographs that had previously undergone animal-presence review and species identification. Silver pheasant (*Lophura nycthemera*) and rhesus macaque (*Macaca mulatta*) images were used as the sample sources because sufficient photographs were available for these species and their images covered a range of forest backgrounds and visible environmental conditions. A total of 102 images were randomly sampled. A subsequent audit of the image-to-species mapping identified 50 silver pheasant images and 52 rhesus macaque images. The images represented 32 scene groups defined from repeated camera viewpoints; 7 groups contained both species and 25 contained only one of the two species.

The visible habitat attributes in each image were verified separately by five researchers: Xiaoming Mo, Yanyan Zhao, Hui Gao, Yang Luo and Wenhua Li. The annotation team included researchers with backgrounds in ecology and computer science. Each reviewer completed an individual workbook for all 102 images, and the five sets of judgments were stored separately. The reviewers examined the entire original image and used the same field definitions, allowed values, and judgment criteria specified in the habitat schema. The review interface also displayed the model output, and the individual workbooks were initialized from the previously established reference labels. This exercise is therefore described as independent between-reviewer verification, not as blinded de novo annotation. For the submitted analysis, the retained reference label was checked against the five-reviewer majority for every image and field; the two were identical in all 714 image–field comparisons.

The final model-generated record that passed the format, schema, and logical-consistency checks was compared with the corresponding expert-reference annotation. For scalar categorical and binary fields, an exact match was recorded only when the model-generated value was identical to the expert-reference value. For the multi-label vegetation_layers field, the predicted and reference labels were treated as unordered sets. An exact match required complete set equality. A prediction containing either a missing vegetation layer or an additional vegetation layer was therefore counted as a mismatch. The order of labels within the array did not affect the comparison, and no partial credit was assigned.

Between-reviewer agreement was calculated from the five separate review files before combining their judgments. Fleiss’ kappa was used for each scalar categorical or binary field. The vegetation_layers arrays were canonicalized as unordered sets and treated as nominal complete-set categories for the primary kappa calculation; supplementary per-layer binary kappas were also calculated for tree, shrub, herb and vine. Pairwise exact agreement and the number of images with unanimous ratings were reported to aid interpretation. When all reviewers used only one category, kappa was reported as not estimable because expected agreement had zero variance, while observed agreement was still reported.

Field-level exact agreement was calculated as the proportion of evaluated images for which the model output exactly matched the corresponding expert-reference annotation. Because overall exact agreement can be affected by class imbalance, additional class-sensitive analyses were conducted for the water-related fields, for which the validation subset contained informative positive and negative or multiclass reference examples. For these fields, balanced accuracy, macro-averaged F1, class support, precision, recall, and F1 were reported. Wilson 95% confidence intervals were calculated for the overall exact-agreement estimate of all seven fields.

To examine performance under different image-acquisition conditions, the 102 images were manually classified as daytime or nighttime from the recorded scene rather than from the camera timestamp, because the embedded timestamps were not reliable indicators of illumination mode. Images showing active infrared illumination, including monochrome rendering, near-field brightening, and a central illumination hotspot, were classified as nighttime images. Images without active infrared illumination were classified as daytime images; this group included one dusk image acquired without supplemental illumination. This procedure identified 93 daytime images and 9 nighttime infrared images. Exact agreement was then calculated separately for each group. Owing to the small nighttime subset, this was treated as a descriptive stratified analysis rather than a formal hypothesis test.

For fields with severely imbalanced reference distributions, the class distribution was explicitly described so that a high overall exact-agreement value would not be interpreted as evidence of reliable recognition for unrepresented categories. In particular, results for visible human disturbance were interpreted separately because the validation subset contained no disturbance-positive expert-reference images.

As a descriptive secondary analysis, field-level exact agreement and expert-reference class distributions were summarized separately for the two species-associated image subsets. This analysis was not used to infer species-specific habitat use or a species effect on model performance. The species and camera-scene composition were confounded: most scene groups contained only one species, and one rhesus-macaque-only scene contributed 23 images.

All disagreements for terrain (*n* = 8) and bare-rock visibility (*n* = 10) were also subjected to a focused visual review. Each terrain disagreement was assigned one primary visually observable ambiguity category. For bare-rock visibility, the review recorded the direction of disagreement, scene grouping, and recurrent visual characteristics. These categories describe the reviewed images; they do not establish causal mechanisms or independent error rates because repeated images from the same viewpoint are not independent.

All 102 images ultimately produced valid structured records after the parsing, validation, and retry procedures described in Section 2.3 and Section 2.4. Therefore, no image was excluded from the field-level analysis, and the valid sample size was 102 for all seven fields.

Passing the structural validation procedure indicated only that a record satisfied the predefined format, schema, and logical-consistency requirements. It did not indicate that the semantic field values were correct. Semantic agreement was evaluated only through comparison with the expert-reference annotations.

The validation was designed as a proof-of-concept assessment under the current sample conditions. The resulting agreement estimates should not be interpreted as definitive measures of generalization across camera models, imaging conditions, camera sites, seasons, ecosystems, or geographic regions.

## 3. Results

### 3.1. Qualitative Examination of Structured Habitat Attribute Outputs

The workflow generally generated complete and interpretable records for the seven predefined habitat fields. Representative outputs contained standardized field names and values corresponding to terrain, bare-rock visibility, vegetation layers, vegetation density, water visibility, water type, and visible human disturbance. The structural validity of these records was determined programmatically as described in Section 2.4.

For images with clearly visible environmental features, the generated records generally showed apparent correspondence with the depicted scene. For example, images containing visually identifiable surface water were commonly assigned water_visible = true together with a corresponding water_type value. Images without visually identifiable water were generally assigned water_visible = false and water_type = none. Similarly, representative forest images produced combinations of vegetation-layer and vegetation-density categories corresponding to the vegetation structure visible in the image.

For fields that included an uncertain category, the model could return this category when the available visual evidence was insufficient for a more definite assignment. Agreement under daytime and nighttime infrared imaging conditions was evaluated separately in Section 3.3.

**Figure 2:**
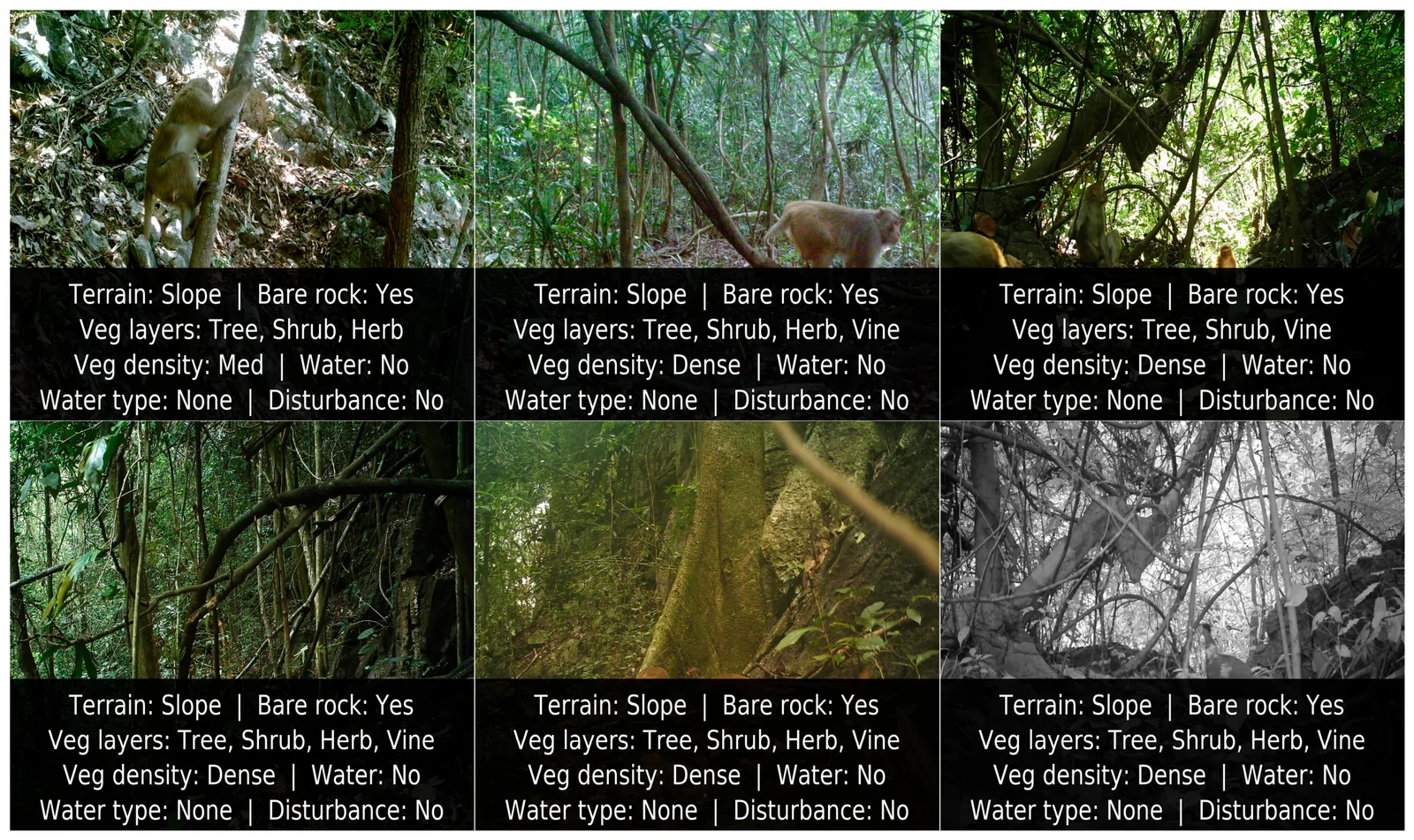
Representative camera-trap images and their corresponding vision–language-model-generated structured habitat records. The examples illustrate the output format and field interpretation and were not used as an independent quantitative evaluation.

Figure 2 presents representative images and their corresponding model-generated structured records. These examples illustrate the form and interpretability of the output but should not be considered an independent assessment of model performance. Quantitative agreement with the expert-reference annotations was evaluated separately in Section 3.2.

### 3.2. Field-Level Exact Agreement with Expert-Reference Annotations

The final valid structured records were compared with the expert-reference annotations for all 102 validation images. Field-level exact agreement was calculated for each of the seven habitat attributes, and the results are presented in Table 2.

The reference distributions were strongly imbalanced for several fields. For terrain, the expert-reference labels comprised 87 slope, 8 steep, 4 uncertain, and 3 flat images. Bare rock was visible in 96 of 102 images. Vegetation density was labeled dense in 99 images and medium in 3 images, with no sparse or uncertain examples. All 102 images were negative for visible human disturbance.

Exact agreement ranged from 90.20% to 99.02% across the seven evaluated fields. Vegetation_density achieved 98.04% exact agreement, followed by water_visible at 96.08%, and vegetation_layers and water_type at 95.10%. Terrain and bare_rock_visible achieved exact-agreement values of 92.16% and 90.20%, respectively.

Human_disturbance_visible produced 99.02% exact agreement. All 102 expert-reference annotations for this field were false, and the model assigned one false-positive disturbance label.

The reference distribution for water_visible was more informative, comprising 82 images without visible water and 20 images with visible water. The model correctly classified all 20 water-positive images and 78 of the 82 water-negative images. This corresponded to an exact agreement of 96.08% (95% CI: 90.35–98.46%), a balanced accuracy of 97.56%, and a macro-F1 score of 94.20%. The majority category accounted for 80.39% of the reference images.

For water_type, the expert-reference annotations comprised 82 none, 10 pond, 9 stream, and 1 uncertain cases. No wetland case was present. Overall exact agreement was 95.10% (95% CI: 89.03–97.89%), balanced accuracy was 96.28%, and macro-F1 was 92.33%. Among the 20 images for which water_visible was true, the model exactly matched the expert water-type annotation in 19 cases, including 9 of 10 pond cases, all 9 stream cases, and the single uncertain case. Class-level precision, recall, and F1 scores for the water-related habitat attributes are presented in Table 3.

### 3.3. Daytime and Nighttime Stratified Agreement

The validation subset contained 93 daytime images acquired without active infrared illumination and 9 nighttime images acquired with infrared illumination. Table 4 reports exact agreement separately for the two imaging groups.

All nine nighttime images matched the expert reference for terrain, bare-rock visibility, water visibility, water type, and visible human disturbance. Eight matched for vegetation layers and seven matched for vegetation density. In the daytime subset, exact agreement ranged from 89.25% for bare-rock visibility to 100.00% for vegetation density.

### 3.4. Between-Reviewer Agreement in Expert Verification

The five separate expert verification files contained complete ratings for all 102 images and seven fields. Fleiss’ kappa was 0.885 for terrain, 1.000 for bare-rock visibility, 0.974 for the exact vegetation-layer set, 1.000 for vegetation density, 0.988 for water visibility, and 0.971 for water type (Table 5). For visible human disturbance, all 510 ratings were false. Observed agreement was therefore 100%, but kappa was not estimable because the ratings contained no between-category variation.

Across the fields, 12 image–field combinations on 11 unique images had at least one differing rating. Terrain accounted for seven of these images, whereas bare-rock visibility had no between-reviewer disagreement. For every image and field, the five-reviewer majority matched the retained expert-reference label. Image-level vote patterns and supplementary per-layer vegetation kappas are provided in the Supplementary Materials.

### 3.5. Species-Associated Descriptive Results

The audited mapping assigned 50 images to silver pheasant and 52 to rhesus macaque. Table 6 reports field-level exact agreement within these two image subsets. Silver-pheasant images had 96.00% terrain agreement and 100.00% bare-rock agreement, whereas rhesus-macaque images had 88.46% and 80.77%, respectively. Agreement for the other five fields ranged from 90.38% to 100.00% in the macaque subset and from 96.00% to 100.00% in the silver-pheasant subset.

The 102 images formed 32 scene groups. Seven groups contained images of both species, whereas 25 were single-species groups. One rhesus-macaque-only group contained 23 images. The expert-reference distributions also differed between the subsets. For example, visible water occurred in 8 of 50 silver-pheasant images and 12 of 52 rhesus-macaque images; expert-visible bare rock occurred in 48 images in each subset.

### 3.6. Focused Review of Terrain and Bare-Rock Disagreements

Focused visual review covered all eight terrain disagreements and all ten bare-rock disagreements. Five terrain disagreements had a limited or fragmented view of the ground plane combined with vegetation or scene clutter, two had perspective or depth ambiguity, and one was acquired under low illumination. Six of the eight terrain disagreements were in rhesus-macaque images and two were in silver-pheasant images.

All ten bare-rock disagreements had the same direction: the expert reference recorded visible rock and the model recorded no visible rock. All ten were rhesus-macaque images from the same scene group. The reviewed images repeatedly showed partial or low-contrast rock exposure within a common viewpoint, together with variable obstruction by animals, vegetation, or a fallen trunk and dappled illumination. Thus, the ten records constitute a concentrated repeated-scene error pattern, not ten independent camera-site failure contexts. The complete image identifiers and review notes are provided in the Supporting Data File; the two selected same-site qualitative comparisons requested by the reviewer are presented separately in the Supplementary Materials.

## 4. Discussion

### 4.1. The Potential of Camera Trap Photographs as Carriers of Microhabitat Information

The core significance of this study lies in extending the functional role of camera trap photographs from simply recording animal occurrences to serving as scene data carriers that simultaneously record the visible microhabitat conditions present when animals appear. For a long time, the primary use of camera traps in ecology has focused on species records, activity rhythm analysis, community composition studies, and population monitoring [1,2,3,4,5,6,33,34], while the large amount of environmental information in image backgrounds has rarely been systematically used. In fact, every camera trap photograph records not only the ecological event of animal occurrence but also visual cues of the local environment at that moment, including terrain, vegetation structure, bare rock exposure, water presence, and traces of human disturbance [16]. Compared with many environmental variables that rely on external spatial data or periodic manual surveys, such photograph-level environmental information has stronger temporal synchronization and contextual relevance [16,18].

This temporal synchronization is particularly important. Traditional habitat surveys usually rely on quadrat measurements or supplementary collection of environmental factors, and there is often a time gap between the survey record and the actual time at which the animal appeared [14,18]. This makes it difficult to accurately reflect the immediate scene conditions when the animal was photographed. By contrast, the background information in camera trap photographs is naturally synchronous with the animal record, which gives it unique value in microhabitat research [16]. Especially under conditions involving short-term environmental changes, such as the formation of standing water after rainfall, changes in surface moisture, or seasonal fluctuations in vegetation cover, the scene information preserved in photographs can supplement those that are difficult to capture using conventional static environmental variables [16,18]. A selected same-site example in the Supplementary Materials illustrates this potential but, without synchronized environmental measurements, does not validate temporal-change detection. Camera-trap photographs should therefore be understood as low-cost, repeatedly accumulated units of visible-scene observation as well as evidence of animal occurrence.

### 4.2. Preliminary Feasibility of Structured Habitat Extraction Based on a Vision–Language Model

The primary technical contribution of this study is the design and proof-of-concept evaluation of a structured post-acquisition processing workflow for converting heterogeneous camera-trap imagery into predefined, machine-readable environmental records. The workflow combines a fixed habitat schema, constrained vision–language-model generation, programmatic parsing, schema validation, logical-consistency checking, automatic retry handling, and traceable storage of the original and parsed outputs. This combination addresses a practical limitation of existing camera-trap archives: environmental information is recorded in the images but is not normally available in a standardized form for subsequent analysis.

The field-level results provide preliminary evidence that a general-purpose vision–language model can assign several visible habitat attributes under the conditions represented in the validation subset. Exact agreement ranged from 90.20% to 99.02% across the seven fields. The separate expert verification files also showed high between-reviewer consistency: all six estimable field-level kappas were at least 0.885, and the retained reference agreed with the five-reviewer majority for every image and field. Terrain produced the lowest kappa and all seven of its between-reviewer disagreement cases, whereas bare-rock visibility was unanimous despite being the lowest-agreement model field. This distinction suggests that the model’s concentrated bare-rock errors were not explained by disagreement among the five reviewers. These reliability results quantify consistency within the present verification workflow; they do not replace a blinded de novo annotation study because reviewers could see the model output and began from pre-populated reference labels. The water-related fields provided the most interpretable evidence because their reference data included both water-positive and water-negative images as well as several represented water types. All 20 water-positive images were correctly identified, and 19 of these images received the same water-type annotation as the expert reference. These results support preliminary feasibility for the represented water categories, although the limited numbers of pond, stream, and uncertain examples and the absence of wetland images prevent comprehensive category-level validation.

The remaining fields require more cautious interpretation than their overall agreement values alone suggest. The expert reference contained only 6 bare-rock-negative images, 3 medium-density images and no sparse or uncertain vegetation-density images. Terrain was also dominated by the slope class (87 of 102 images). These distributions make the current data useful for identifying common failure patterns but insufficient for estimating robust class-specific performance across the full schema.

Focused review refined the interpretation of the two lowest-agreement fields. Five of eight terrain disagreements involved a limited or fragmented ground-plane view with scene clutter, two involved perspective or depth ambiguity, and one occurred under low illumination. All ten bare-rock disagreements were false negatives concentrated at a single repeated camera viewpoint, where exposed rock was partial or low-contrast and variably obscured. This concentration indicates a site-specific visual failure pattern and shows why image-level counts should not be treated as independent site-level evidence.

The species-associated subset results require the same caution. The silver-pheasant subset showed higher descriptive agreement for terrain and bare-rock visibility than the rhesus-macaque subset, but only 7 of 32 scene groups contained both species and one macaque-only scene contributed 23 images, including all ten bare-rock disagreements. The observed differences therefore cannot be separated from camera-scene composition and must not be interpreted as species effects, habitat selection, or differential model capability by animal species.

Differences in agreement among the fields also highlight the importance of visual observability and category definition. Terrain and bare-rock visibility produced lower pooled exact-agreement values than vegetation density and the water-related fields. In the descriptive imaging-mode analysis, however, all nine nighttime infrared images matched the reference for terrain and bare-rock visibility, whereas vegetation layers and vegetation density showed one and two nighttime mismatches, respectively. The present data therefore do not support a general claim that infrared images were more difficult across all fields. The small nighttime subset and differences in category composition prevent causal attribution of the observed subgroup differences to illumination mode. Future schema development should consider both ecological relevance and whether each attribute can be interpreted consistently under the available imaging conditions.

Although human_disturbance_visible produced the highest numerical exact agreement, this result was based entirely on disturbance-negative expert-reference images. It therefore reflects agreement in the negative class and does not demonstrate the ability to detect visible human disturbance. This example illustrates why overall exact agreement must be interpreted together with the reference-class distribution and why future validation datasets should deliberately include both positive and negative examples for binary attributes.

The extracted fields should be interpreted as model-inferred visible scene attributes rather than direct physical measurements. For example, water_visible records whether water can be visually identified in an image; it does not measure water depth, discharge, soil moisture, or hydrological state. Similarly, vegetation_density represents an image-based categorical interpretation and is not equivalent to field-measured canopy cover, biomass, or remotely sensed vegetation indices. Terrain describes the visible topographic appearance within the camera view and is not equivalent to a surveyed slope or digital elevation measurement. This distinction is important when integrating image-derived records with measurements from conventional environmental sensors, field surveys, or remote-sensing products.

The proposed workflow should therefore be regarded as a complementary method for extracting standardized information already present in camera-trap images. It does not introduce a new camera sensor or a new vision–language-model architecture, nor does it replace direct environmental measurements. Its methodological value lies in structuring previously underused image content and providing a traceable process through which this information can be parsed, checked, reviewed, and evaluated.

### 4.3. Application Value in Karst Forest Scenes

This study used Nonggang Reserve as a proof-of-concept site because of its methodological suitability. The karst region of southern China is geomorphologically complex, and environmental elements are highly spatially heterogeneous [19,22,25,29,35,36]. Many key habitat characteristics occur at fine spatial scales that are difficult for remote sensing products to resolve effectively [22,35,36]. In such environments, relying solely on macro-topographic or land-cover data often fails to accurately reflect the immediate microhabitat conditions associated with animal activity [16,17,35,36,37].

Camera trap photographs provide a ground-level observational medium for recording these fine-scale scene features at the time of animal occurrence [16]. By automatically extracting visible environmental cues in photographs into structured attributes, the method proposed in this study offers a new technical pathway for microhabitat characterization in karst forests. Compared with traditional manual surveys, this method does not require additional synchronized field habitat measurements for each animal record and may therefore reduce the additional field effort required for image-level habitat characterization in long-term monitoring programs [14]. Compared with coarser remote-sensing products, photograph-level habitat records provide a ground-level perspective that can capture visible understory and ground-surface features that may not be resolved at broader spatial scales [16].

Furthermore, the selected paired examples in the Supplementary Materials suggest that the method may reflect temporal differences in visible microhabitat conditions across both shorter and longer intervals, rather than only describing static background features. If confirmed through systematic temporal validation, this capability could be particularly useful in karst forests, where local moisture conditions, exposed ground-surface states, and understory scene structures may vary in response to rainfall, drought, and seasonal transitions [22,35,36]. If the structured environmental records extracted from camera trap photographs can be further integrated with temporal information, site information, and meteorological processes in future research, it may become possible to build a microhabitat monitoring framework with higher temporal resolution [16,18,37]. Such a framework could provide a new observational scale for understanding relationships between animal activity and environmental change in complex mountain ecosystems.

### 4.4. Limitations and Future Directions

Several limitations should be considered when interpreting this proof-of-concept study. The validation dataset comprised 102 animal-containing images associated with two species from a single protected area and therefore does not represent the full range of backgrounds, ecosystems, seasons, camera configurations, or geographic conditions encountered in broader camera-trap networks. Several attribute categories were also imbalanced or absent, including disturbance-positive and wetland scenes, and only nine nighttime infrared images were available. Accordingly, performance for underrepresented categories and imaging conditions requires validation using larger, stratified datasets covering additional sites, seasons, ecosystems, and both animal-containing and animal-free images.

The evaluation design also has methodological limitations. Although five experts completed separate verification files, the process was not fully blinded de novo annotation because model outputs were visible and the files were initialized with prior reference labels. The resulting between-reviewer agreement should therefore be interpreted as reliability within this verification procedure. In addition, the study evaluated a single general-purpose vision–language model within one structured extraction workflow and did not assess run-to-run variation among valid outputs, alternative models, or the separate contributions of prompting, schema constraints, and rule-based validation. These comparisons were outside the scope of the present feasibility assessment but would be valuable in future benchmarking studies.

Finally, the extracted attributes represent only environmental characteristics visible within the camera field of view. The same-site image pairs illustrate differences in visually apparent scene conditions but do not establish temporal-change detection without independent environmental measurements. Future work should therefore combine broader and more balanced validation with fully blinded expert annotation and repeated inference. Comparative model and workflow-component analyses, together with integration of image-derived attributes with weather, topographic, remote-sensing, and direct environmental measurements, could further evaluate the robustness and ecological utility of the proposed approach.

## 5. Conclusions

This study developed and evaluated a vision–language-model-based workflow for converting environmental information in camera-trap images into seven structured visible habitat attributes. The workflow combined schema-constrained prompting, structured JSON generation, programmatic parsing, logical-consistency validation, automatic retry handling, and traceable output storage. All 102 validation images ultimately produced valid structured records, and field-level exact agreement with expert-reference annotations ranged from 90.20% to 99.02%. Five separate expert-verification files yielded Fleiss’ kappa values of 0.885–1.000 for the six estimable fields; visible human disturbance had 100% observed agreement but no category variation, so kappa was not estimable. The retained reference matched the five-reviewer majority in all image–field comparisons.

The water-related fields provided the most informative evidence because their reference data included both positive and negative images and several represented water types. All 20 water-positive images were correctly identified, and 19 received the same water-type annotation as the expert reference. By contrast, the high agreement for visible human disturbance was based entirely on disturbance-negative reference images and should not be interpreted as evidence of disturbance-detection capability. These findings demonstrate the importance of interpreting overall agreement together with reference-class distributions.

The results provide preliminary evidence that environmental information already captured by camera-trap imaging sensors can be transformed into standardized image-level records. These records may complement, but should not be treated as replacements for, field measurements, dedicated environmental sensors, or remote-sensing products. Because the evaluation was limited to a small, single-region dataset with imbalanced category distributions, broader validation, fully blinded de novo annotation, and repeated-inference analysis are required before the workflow can be considered generalizable. Model comparison and prompt or schema ablation would be required for benchmark or component-attribution claims, which are outside the scope of this study. Nevertheless, the study establishes a traceable proof-of-concept framework for the structured reuse of environmental background information in camera-trap image archives.

## Figures and Tables

**Table 1:** Field definitions, data types, allowed values, and validation constraints for the structured output of visible habitat attributes in camera trap photographs.

Field	Data Type	Allowed Values/Description	Notes & Constraints
terrain	string	flat, slope, steep, uncertain	-
bare_rock_visible	Boolean	true/false	Whether exposed rock is visible
vegetation_layers	array [string]	Any combination of tree, shrub, herb, vine (can be empty)	Observed vertical vegetation layers; multiple selection allowed
vegetation_density	string	dense, medium, sparse, uncertain	Overall vegetation cover/density estimation
water_visible	Boolean	true/false	Whether water body is observed
water_type	string	none, stream, pond, wetland, uncertain	Must be ‘none’ when water_visible = false
human_disturbance_visible	Boolean	true/false	Presence of obvious human disturbance traces

**Table 2:** Field-level exact agreement between vision–language-model outputs and expert-reference annotations in the validation subset (*n* = 102).

Field	Exact Matches	Valid Samples	Exact Agreement (%)	Wilson 95% CI
terrain	94	102	92.16	85.28–95.97
bare_rock_visible	92	102	90.20	82.89–94.59
vegetation_layers	97	102	95.10	89.03–97.89
vegetation_density	100	102	98.04	93.13–99.46
water_visible	98	102	96.08	90.35–98.46
water_type	97	102	95.10	89.03–97.89
human_disturbance_visible	101	102	99.02	94.65–99.83

**Table 3:** Class-level precision, recall, and F1 score for water-related habitat attributes in the validation subset.

Field	Expert Class	Support	Predicted Support	Precision (%)	Recall (%)	F1 (%)
water_visible	false	82	78	100.00	95.12	97.50
water_visible	true	20	24	83.33	100.00	90.91
water_type	none	82	78	100.00	95.12	97.50
water_type	pond	10	10	90.00	90.00	90.00
water_type	stream	9	13	69.23	100.00	81.82
water_type	uncertain	1	1	100.00	100.00	100.00

**Table 4:** Descriptive field-level exact agreement and Wilson 95% confidence intervals for daytime and nighttime infrared images.

Field	Daytime, *n* = 93	Nighttime Infrared, *n* = 9
terrain	85/93, 91.40% (83.93–95.58)	9/9, 100.00% (70.09–100.00)
bare_rock_visible	83/93, 89.25% (81.33–94.05)	9/9, 100.00% (70.09–100.00)
vegetation_layers	89/93, 95.70% (89.46–98.31)	8/9, 88.89% (56.50–98.01)
vegetation_density	93/93, 100.00% (96.03–100.00)	7/9, 77.78% (45.26–93.68)
water_visible	89/93, 95.70% (89.46–98.31)	9/9, 100.00% (70.09–100.00)
water_type	88/93, 94.62% (88.03–97.68)	9/9, 100.00% (70.09–100.00)
human_disturbance_visible	92/93, 98.92% (94.16–99.81)	9/9, 100.00% (70.09–100.00)

**Table 5:** Between-reviewer agreement across five separate expert-verification files for 102 images. Pairwise agreement is the mean across the ten reviewer pairs.

Field	Fleiss’ Kappa	Mean Pairwise Exact Agreement (%)	Unanimous Images, *n* (%)	Images with Disagreement
terrain	0.885	96.86	95 (93.14)	7
bare_rock_visible	1.000	100.00	102 (100.00)	0
vegetation_layers (exact set)	0.974	99.22	100 (98.04)	2
vegetation_density	1.000	100.00	102 (100.00)	0
water_visible	0.988	99.61	101 (99.02)	1
water_type	0.971	99.02	100 (98.04)	2
human_disturbance_visible	Not estimable	100.00	102 (100.00)	0

**Table 6:** Descriptive exact agreement by animal-associated image subset. These values are not estimates of species effects because species and scene composition were confounded.

Field	Silver Pheasant, *n* = 50	Rhesus Macaque, *n* = 52
terrain	48/50 (96.00%)	46/52 (88.46%)
bare_rock_visible	50/50 (100.00%)	42/52 (80.77%)
vegetation_layers	48/50 (96.00%)	49/52 (94.23%)
vegetation_density	48/50 (96.00%)	52/52 (100.00%)
water_visible	50/50 (100.00%)	48/52 (92.31%)
water_type	50/50 (100.00%)	47/52 (90.38%)
human_disturbance_visible	50/50 (100.00%)	51/52 (98.08%)

## Data Availability

The de-identified image-level vision–language-model outputs, retained expert-reference annotations, five separate expert-verification files, between-reviewer agreement summary, disagreement vote patterns, and calculation script are provided in the Supplementary Materials. The corresponding 102 camera-trap photographs are available to the journal editors and peer reviewers for confidential assessment during the peer-review process but cannot be made publicly available because they originate from wildlife monitoring in protected areas and may contain sensitive monitoring information. Following publication, access to these photographs may be granted by the corresponding author upon reasonable request, subject to approval by the relevant protected-area management authority and, where appropriate, completion of a data-use agreement.

## Supplementary Materials

The following supporting information can be downloaded at: https://www.mdpi.com/article/10.3390/s26196240/s1, Figure S1: Same-site comparison over a 9-day interval; Figure S2: Same-site comparison over a 76-day interval; Table S1: Focused review of terrain disagreements; Table S2: Focused review of bare-rock disagreements; Table S3: Between-reviewer agreement for the seven retained habitat fields; expert_reference_and_model_outputs.xlsx: expert-reference annotations and corresponding model outputs; annotation_disagreements.csv: non-unanimous image-field combinations; IAA_summary.csv: between-reviewer agreement summary; Expert_01.xlsx–Expert_05.xlsx: anonymized expert-verification files; calculate_iaa.py: script used to calculate between-reviewer agreement.
