# Supplementary material for: Structured Habitat Attribute Extraction from Camera-Trap Images Using a Vision–Language Model: A Proof-of-Concept Study in a Karst Forest
Source: Sensors (Basel). 2026 Oct 1;26(19):6240. doi: 10.3390/s26196240 (PMC13667531; doi:10.3390/s26196240)
Supplement: Supplementary file 1 [file sensors-26-06240-s001.zip › Public_Supplementary_Materials/Supplementary_Material.pdf]

Supplementary Material: Same-Site Comparisons, Error Review, and Between-Reviewer Agreement

S1. Scope

Two image pairs acquired from the same camera viewpoints at different times were selected to illustrate whether model-generated structured records reflected visually apparent scene differences. These examples were not included as an independent quantitative validation and do not establish temporal-change detection.

S2. Nine-day comparison

The first pair was acquired on 27 May 2017 and 5 June 2017, with an interval of 9 days. The overall background and viewpoint were similar. Visible standing water was present in part of the earlier image, and the model record assigned `water_visible = true`. Visible water was not identified in the corresponding area of the later image, and the record assigned `water_visible = false`. No synchronized rainfall, hydrological, or soil-moisture measurements were available, so the cause of the visual difference cannot be determined.

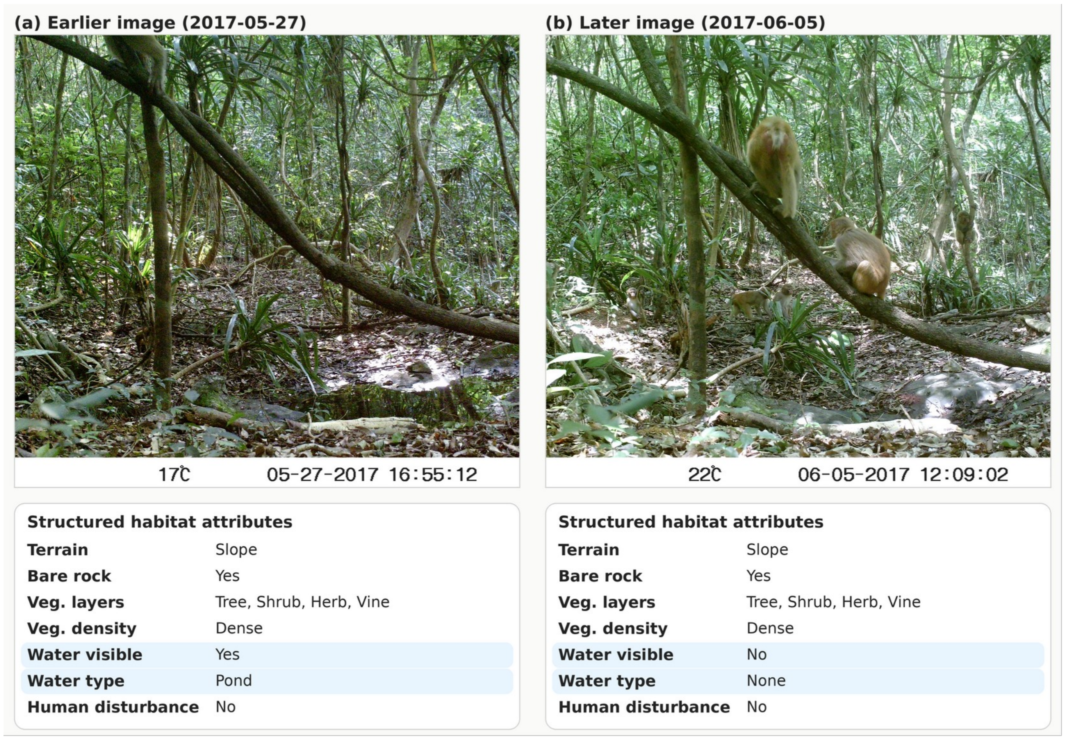

Figure S1. Illustrative same-site comparison over a 9-day interval. The model-generated records reflected a visible difference in surface-water presence. This selected pair is qualitative and does not constitute validation of temporal-change detection.

S3. Seventy-six-day comparison

The second pair was acquired on 12 February 2017 and 29 April 2017, with an interval of 76 days. Because of the longer interval, the pair represents a multi-week or within-season difference in visible scene conditions rather than immediate short-term change.

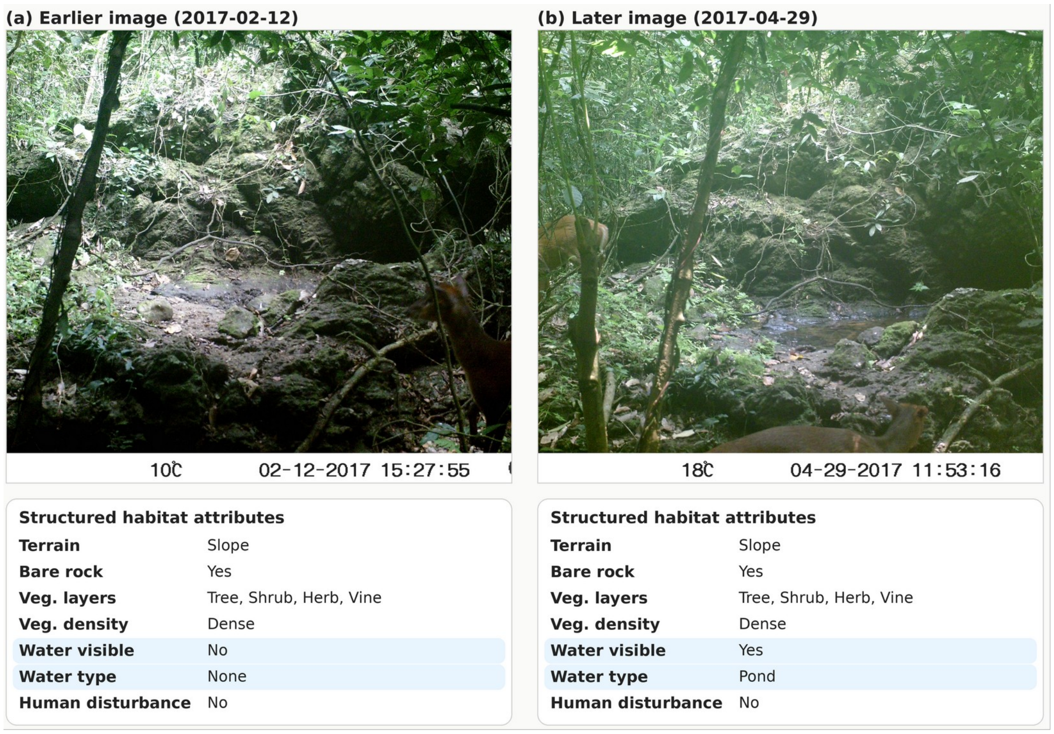

Figure S2. Illustrative same-site comparison over a 76-day interval. The pair demonstrates that model-generated records from a common viewpoint can differ when visible scene conditions differ.

S4. Interpretation limit

The examples were selected for illustration and were not evaluated against independently measured rainfall, hydrology, vegetation, or other environmental data. Formal temporal monitoring would require a predefined longitudinal sample, repeated same-site observations, independently established change labels, and synchronized environmental measurements.

S5. Image-level review of terrain and bare-rock disagreements

Table S1. All terrain disagreements and the primary observable ambiguity category assigned during focused visual review.

Image MD5	Model / expert	Scene	Primary observable ambiguity
00000e8536f64fac9 4c344a03661cb20	slope / flat	1	Perspective or depth ambiguity
0866b52849d40b3e	uncertain / slope	4	Limited or

e4504ea0fb1c9075			fragmented ground plane with scene clutter
0b5d2921bc2747c56 e02a8cc08b723c4	uncertain / slope	4	Limited or fragmented ground plane with scene clutter
196afd2026ccce7ad 2a5c7d305332638	uncertain / slope	4	Limited or fragmented ground plane with scene clutter
1e1c33ab3ca231393b 681f926cce2b06	uncertain / flat	15	Limited or fragmented ground plane with scene clutter
1ee20ed84d7f4f004 56c11a033e29aac	uncertain / flat	17	Limited or fragmented ground plane with scene clutter
207f914556883fa78 cdbd546872d0699	slope / steep	16	Perspective or depth ambiguity
45ac4c23264920344 b1015de3c66d50e	flat / uncertain	25	Low illumination

Table S2. All bare-rock disagreements. Every record was a false negative (model `false`, expert `true`) from rhesus-macaque-associated scene group 4.

Image MD5	Recurrent visual pattern
02fa0f42c449ff28f828c806ec64cb8a	Partial or low-contrast rock exposure with variable scene occlusion
054c8392c2432f906ab48db03c188ba2	Same repeated-viewpoint pattern
06ae63bb472827c9a202d0d86db1f1b8	Same repeated-viewpoint pattern
0866b52849d40b3ee4504ea0fb1c9075	Same repeated-viewpoint pattern
09de0db0523b1eb6bee960a1261ddbc0	Same repeated-viewpoint pattern
196afd2026ccce7ad2a5c7d305332638	Same repeated-viewpoint pattern
2571677d17d263b90aef34b26b2fa870	Same repeated-viewpoint pattern
37ea550b55c68d43e6b9c7c77bc7776c	Same repeated-viewpoint pattern
40ff32621cb1b6336da29c11d74f5468	Same repeated-viewpoint pattern
47181bfe2e07413a843450568771bafc	Same repeated-viewpoint pattern

Because Table S2 consists of repeated images from one scene group, the ten image-level records should not be interpreted as ten independent camera-site failure contexts.

S6. Independent expert verification and between-reviewer agreement

Five experts (Xiaoming Mo, Yanyan Zhao, Hui Gao, Yang Luo and Wenhua Li) completed separate verification workbooks covering the same 102 images and seven habitat fields. The interface displayed the AI result, and the files were initialized with the prior reference values. The exercise is therefore evidence of independent between-reviewer verification, not a blinded de novo annotation experiment.

Fleiss' kappa was calculated across the five ratings for each field. For `vegetation_layers`, arrays were canonicalized as unordered complete sets for the primary nominal-category calculation. Pairwise exact agreement is the mean across the ten reviewer pairs.

Table S3. Between-reviewer agreement across five separate expert-verification files for 102 images.

Field	Fleiss' kappa	Pairwise agreement range (%)	Mean pairwise agreement (%)	Unanimous images, n (%)	Disagreement images
terrain	0.885	94.12–99.02	96.86	95 (93.14)	7
bare_rock_visible	1.000	100.00–100.00	100.00	102 (100.00)	0
vegetation_layers (exact set)	0.974	98.04–100.00	99.22	100 (98.04)	2
vegetation_density	1.000	100.00–100.00	100.00	102 (100.00)	0
water_visible	0.988	99.02–100.00	99.61	101 (99.02)	1
water_type	0.971	98.04–100.00	99.02	100 (98.04)	2
human_disturbance_visible	Not estimable	100.00–100.00	100.00	102 (100.00)	0

Kappa for `human_disturbance_visible` is not estimable because every one of the 510 ratings was `false`, leaving no between-category variation. This is not a failure of observed agreement; all five reviewers agreed on all 102 images.

Table S4. Per-layer binary agreement for `vegetation_layers`.

Layer
tree
shrub
herb

vine

Across all seven fields, 12 image-field combinations on 11 unique images contained at least one differing rating. The complete vote patterns are listed in `annotation_disagreements.csv`. Terrain contained seven disagreement images, while bare-rock visibility contained none. The five-reviewer majority matched the retained expert-reference label in all 714 image-field comparisons. No post hoc adjudication claim is made; this result is a majority-verification check of the retained reference.
